# Real-World Effectiveness of Dupilumab in Severe Uncontrolled Asthma: Clinical Remission on Treatment, Predictors of Complete Response and Impact on Type-2 Comorbidities

**DOI:** 10.3390/jcm15166429

**Published:** 2026-08-20

**Authors:** Eusebi Chiner, Ignacio Boira, Mónica Antón, María Ángeles Bernabeu, José Luis Lafuente, María Pignatelli, Pilar Soro, Violeta Esteban, Paula Fernández-Martínez, Anays Martínez-Gómez

**Affiliations:** 1 Pneumology Department, San Juan de Alicante University Hospital, Ctra N-332, s/n, 03550 San Juan de Alicante, Alicante, Spain; echinervives@gmail.com (E.C.); mariaapignatellii@gmail.com (M.P.); violeta_er@hotmail.com (V.E.); paulafernandez1441@gmail.com (P.F.-M.); docanays@gmail.com (A.M.-G.); 2 Department of Clinical Medicine, Miguel Hernández University, 03202 Elche, Alicante, Spain; 3 Allergy Department, San Juan de Alicante University Hospital, 03550 San Juan de Alicante, Alicante, Spain; manton.girones@hotmail.com; 4 Pharmacy Department, San Juan de Alicante University Hospital, 03550 San Juan de Alicante, Alicante, Spain; bernabeu_marmar@gva.es; 5 Otorhinolaryngology Department, San Juan de Alicante University Hospital, 03550 San Juan de Alicante, Alicante, Spain; lafuente_jos@gva.es; 6 Dermatology Department, San Juan de Alicante University Hospital, 03550 San Juan de Alicante, Alicante, Spain; soro_pilmar@gva.es

**Keywords:** severe asthma, uncontrolled severe asthma, dupilumab, biologic therapy, clinical remission, FEOS, EXACTO, rhinosinusitis with nasal polyps, atopic dermatitis

## Abstract

**Background/Objectives**: Real-world evidence on dupilumab-induced clinical remission on treatment and on the predictors of complete response in severe uncontrolled asthma (SUA) is still limited. **Methods**: We conducted a single-centre ambispective uncontrolled observational study including 152 adults with SUA who started dupilumab. Lung function, type-2 (T2) biomarkers, patient-reported outcomes, exacerbations, oral corticosteroid (OCS) use, emergency-department (ED) visits and hospitalisations were compared between baseline and last follow-up. Response was graded with FEOS and EXACTO. Independent predictors of complete response (EXACTO = 1) were explored by multivariable logistic regression. **Results**: Mean age was 50 ± 12 years, 67.1% were women, baseline FEV1 was 82 ± 22% predicted, blood eosinophils were 612 ± 577 cells/µL and FeNO was 55 ± 46 ppb. After 21 ± 8 months, ACT improved from 15.7 to 23.3, ACQ-5 from 4.0 to 0.35, mini-AQLQ from 2.18 to 4.53, SNOT-22 from 52 to 16, and FEV1 by 275 ± 180 mL (all *p* < 0.001). Annual exacerbations decreased from 7.9 to 0.7, ED visits from 3.17 to 0.28, hospitalisations from 0.71 to 0.08, and maintenance OCS use from 11.8% to 3.9%. According to EXACTO, 95 patients (62.5%) achieved complete response/super-response. Independent predictors were higher baseline FEV1, higher eosinophil count, allergic rhinitis, rhinosinusitis with nasal polyps and atopic dermatitis, whereas prior OCS bursts and higher FeNO were inversely associated. Model discrimination was adequate (AUC = 0.91). **Conclusions**: Dupilumab achieved clinical remission on treatment in almost two-thirds of patients with SUA and markedly reduced healthcare utilisation. A T2-comorbidity-rich profile identified patients with the highest probability of complete response.

## 1. Introduction

Severe uncontrolled asthma (SUA) affects approximately 3–10% of the asthmatic population but is responsible for most of the disease burden, healthcare expenditure and mortality [1,2]. It is defined by the persistence of symptoms, recurrent exacerbations and/or oral corticosteroid (OCS) dependence despite optimised treatment at GINA steps 4–5 [1,3]. Spanish epidemiological data suggest that 3.9% of asthmatic patients meet criteria for SUA, with a major impact on quality of life and consumption of healthcare resources [4,5].

Approximately 50–70% of cases show a type-2 (T2) inflammatory pattern, characterised by blood eosinophilia, elevated fractional exhaled nitric oxide (FeNO), high total IgE and the frequent presence of T2 comorbidities such as chronic rhinosinusitis with nasal polyps (CRSwNP), allergic rhinitis, atopic dermatitis and eosinophilic oesophagitis [6,7]. Persistent T2 inflammation is associated with airway remodelling, irreversible airflow limitation and a higher exacerbation rate [8,9].

The introduction of biologic therapies has profoundly modified the natural history of severe asthma. Beyond the reduction of exacerbations and OCS exposure, the new clinical goal is to achieve disease remission while on treatment, defined by the absence of symptoms, stable optimised lung function, no exacerbations and no need for maintenance OCSs [3,7]. Dupilumab is a fully human IgG4 monoclonal antibody directed against the α-subunit of the IL-4 receptor (IL-4Rα), which simultaneously blocks the signalling of IL-4 and IL-13, two upstream cytokines that orchestrate IgE class-switching, eosinophil trafficking, goblet-cell metaplasia and airway remodelling [10,11].

The pivotal phase-3 trials LIBERTY ASTHMA QUEST [10] and VENTURE [11] demonstrated that dupilumab reduces severe exacerbations by 46–70%, improves pre-bronchodilator FEV1 by 130–240 mL and allows OCS withdrawal in 70% of glucocorticoid-dependent patients, with the greatest benefit in patients with high baseline eosinophil count and FeNO. Subsequently, dupilumab has been approved for moderate-to-severe atopic dermatitis (LIBERTY AD CHRONOS) [12], CRSwNP (SINUS-24/52) [13], eosinophilic oesophagitis (LIBERTY EoE TREET) [14] and, more recently, for COPD with T2 inflammation (BOREAS, NOTUS) [15,16], making it the first and only cross-organ T2 modulator currently available.

However, real-world cohorts include patients usually excluded from randomised trials—multiple T2 comorbidities, fixed airway obstruction, active smokers, prior failure of other biologics and chronic OCS dependence [17,18]. To standardise the assessment of response in clinical practice, two Spanish-developed scores have gained acceptance: the FEOS score [19], which integrates FEV1, exacerbations, OCS and symptoms, and the multidimensional EXACTO score [20], which stratifies patients into non-response, partial response, good response and complete response/super-response. The use of these scores in real-life cohorts of dupilumab is still scarce.

The aim of the present study was therefore: (i) to evaluate the real-world effectiveness of dupilumab in a cohort of adults with SUA followed at our centre, applying the FEOS and EXACTO scores; (ii) to quantify its impact on T2 comorbidities, OCS consumption and healthcare resource utilisation; and (iii) to identify baseline factors independently associated with a complete response by means of a multivariable logistic regression model.

## 2. Materials and Methods

### 2.1. Study Design and Population

We conducted a single-centre, ambispective, observational, real-world cohort study at the Severe Asthma Unit of San Juan de Alicante University Hospital (Spain) between June 2019 and October 2025. All consecutive adults (≥18 years) with a diagnosis of severe uncontrolled asthma, established according to the GINA and GEMA-5.5 criteria [1,2,3], who started dupilumab were included. Patients had to fulfil one or more of the following: ACT < 20 and/or ACQ-5 ≥ 1.5; ≥2 severe exacerbations requiring systemic corticosteroids in the previous 12 months; ≥1 hospitalisation; or OCS dependence, despite optimised GINA step 4–5 therapy. Treatment adherence was assessed through clinical interview and medication review, and inhaler technique was checked by direct observation during the clinical visit. Baseline controller therapy, including prescribed ICS/LABA and LAMA doses, was recorded. Two patients with hyper-IgE syndrome and a severe asthma phenotype fulfilling the same criteria were also included.

### 2.2. Treatment Protocol and Follow-Up

Dupilumab was administered subcutaneously according to the approved dosing regimen applicable to each patient at treatment initiation. The prescribed dose and dosing regimen were recorded at baseline. As this was an observational real-world study, treatment selection and dosing were determined by the treating physicians according to the applicable prescribing information and routine clinical practice. Background controller therapy (ICS/LABA ± LAMA, leukotriene receptor antagonists, antihistamines) was maintained at baseline; maintenance OCSs were progressively tapered according to a pre-specified protocol after the first 4 months of treatment. Clinical evaluations took place at baseline (T0), 1 month, 4 months and every 6 months thereafter, ensuring assessment of effectiveness, safety and adherence. Because this was an ambispective real-world cohort, the duration of individual follow-up varied according to the date of treatment initiation and the date of the last available clinical assessment. Outcome measures based on healthcare utilisation were annualised to account for differences in observation time, whereas response and remission classifications were based on the available evaluable follow-up assessment.

### 2.3. Variables and Outcomes

Demographic, anthropometric and smoking data were recorded for all patients. T2 comorbidities were recorded from the medical history and confirmed from the patient’s clinical records and, when applicable, specialist assessment.

Allergic rhinitis, atopic dermatitis, NSAID-exacerbated respiratory disease (NERD), food allergy, allergic bronchopulmonary aspergillosis, bronchiectasis, obstructive sleep apnoea and obesity were considered present when a previous established diagnosis was documented. CRSwNP/CRSsNP and anosmia/hyposmia were assessed according to previous otorhinolaryngological evaluation and available clinical records. Comorbidity status was assessed at baseline and changes during follow-up were recorded when clinically documented. Spirometry was performed according to SEPAR and ATS/ERS standards [21]. Spirometry was consistently performed after bronchodilator administration, and after-bronchodilator FEV1 was used for all analyses. T2 biomarkers measured at every visit included blood eosinophil count, total serum IgE and FeNO. Patient-reported outcomes (PROs) were collected at every visit [22]: Asthma Control Test (ACT), 5-item Asthma Control Questionnaire (ACQ-5), 100 mm symptom visual analogue scale (VAS), mini-Asthma Quality-of-Life Questionnaire (mini-AQLQ) and the 22-item Sino-Nasal Outcome Test (SNOT-22).

Healthcare resource utilisation was registered as the number of severe exacerbations, OCS bursts, ED visits and hospitalisations during the 12 months preceding dupilumab and during the follow-up period (annualised). Response was graded using the FEOS score [19] and the multidimensional EXACTO score [3,20], which classifies patients as non-response, partial response, good response or complete response/super-response based on changes in exacerbations, ACT, systemic corticosteroid use and FEV1 relative to T0. Clinical remission on treatment was defined as the simultaneous fulfilment of: (i) no exacerbations, (ii) absence of maintenance OCS, (iii) ACT ≥ 20 or ACQ-5 < 1.5, and (iv) stable or improved lung function during the last 12 months of follow-up.

Safety was assessed at each follow-up visit by recording treatment-emergent adverse events, serious adverse events, and treatment discontinuations, including the reason for discontinuation.

### 2.4. Statistical Analysis

Quantitative variables were assessed for distributional characteristics and are expressed as mean ± standard deviation (SD) for approximately normally distributed variables and as median with interquartile range (IQR) for non-normally distributed variables. Paired comparisons between baseline and post-treatment values were performed using the paired Student’s *t*-test for normally distributed variables and the Wilcoxon signed-rank test for non-normally distributed variables. Categorical variables were compared using the McNemar test for paired data. A multivariable logistic regression model with backward stepwise elimination (removal threshold *p* > 0.10) was built, including variables with *p* < 0.20 in the univariate analysis and ensuring no multicollinearity (variance inflation factor < 3). Model discrimination was assessed by the area under the ROC curve (AUC) and calibration by the Hosmer–Lemeshow test. Analyses were performed with IBM SPSS Statistics v.27 (IBM Corp., Armonk, NY, USA) and Python 3.13 (statsmodels). A two-tailed *p* < 0.05 was considered statistically significant. This study was reported in accordance with the Strengthening the Reporting of Observational Studies in Epidemiology (STROBE) recommendations (Supplementary Material S1).

### 2.5. Ethics

This study was conducted in accordance with the principles of the Declaration of Helsinki. The protocol was approved by the Drug Research Ethics Committee (CEIm) of the University Hospital of Elda (code 2023/04, approval date: 18 April 2023). All participants gave written informed consent prior to inclusion. The authors declare that generative artificial intelligence (Genspark 6.0) was used in the preparation, writing, editing, or revision of this manuscript.

## 3. Results

### 3.1. Baseline Characteristics

A total of 152 patients were included: 102 women (67.1%) and 50 men (32.9%), with a mean age of 50 ± 12 years, BMI of 26.4 ± 5.0 kg/m^2^ and mean follow-up of 21 ± 8 months. Half of the patients had eosinophilic asthma with CRSwNP (50.0%), 18.0% had allergic late-onset asthma, 16.0% had allergic early onset asthma, 8.0% had intrinsic asthma with high IgE and 8.0% had eosinophilic asthma without CRSwNP. Thirty patients (19.7%) had previously received another biologic agent (benralizumab in 27, mepolizumab in two and omalizumab in one). The full baseline profile is summarised in Table 1.

### 3.2. Clinical, Functional and Patient-Reported Outcomes

All efficacy variables improved significantly after dupilumab treatment (Table 2). The ACT increased from 15.7 ± 2.1 to 23.3 ± 1.4 (Δ +7.6, *p* < 0.001), with all evaluable patients reaching the well-controlled threshold of ACT 20 at the last available assessment. The ACQ-5 fell from 4.0 ± 0.9 to 0.35 ± 0.37 (Δ −3.7, *p* < 0.001), well above the minimal clinically important difference (MCID) of 0.5. Quality of life (mini-AQLQ) improved from 2.18 ± 0.36 to 4.53 ± 0.37 (Δ +2.4, *p* < 0.001) and the SNOT-22 improved by −36.6 points (*p* < 0.001), surpassing its MCID of 8.9. FEV1 increased by 275 ± 180 mL (*p* < 0.001) and FeNO decreased by 33 ppb (*p* < 0.001). Olfaction improved in 60% of patients with previous anosmia/hyposmia. Total IgE decreased markedly (1040 → 97 IU/mL, *p* = 0.001), whereas blood eosinophils showed a non-significant minor change (612 → 551 cells/µL, *p* = 0.245), in agreement with the mechanism of action of dupilumab, which does not directly deplete eosinophils [10].

### 3.3. Concomitant Medication, OCS Sparing and Healthcare Utilisation

Use of rescue and add-on therapy decreased significantly after dupilumab (Table 3). Short-acting β2-agonist (SABA) use fell from 96.1% to 21.7%, anticholinergic use from 51.3% to 37.5%, leukotriene receptor antagonist use from 65.1% to 45.4% and antihistamine use from 50.7% to 28.9% (all *p* ≤ 0.002). One-third of patients (32.9%) reduced their inhaled corticosteroid dose. Among the 18 patients receiving maintenance OCSs at baseline, one patient was lost to follow-up and therefore was not evaluable for OCS tapering or withdrawal. Among the remaining 17 evaluable patients, OCSs were tapered in 12/17 (70.6%) and completely withdrawn in 10/17 (58.8%), with a mean dose decrease from 7.0 to 2.3 mg/day prednisone-equivalent (*p* < 0.001). Hospital admissions decreased by 88%, ED visits by 91% and OCS bursts by 91% (all *p* < 0.001). During follow-up, eight patients (5.3%) discontinued dupilumab. Treatment-related adverse events leading to discontinuation included eosinophilic pneumonia in three patients (2.0%) and a flu-like syndrome in one patient (0.7%). No additional serious adverse events leading to treatment discontinuation were recorded.

### 3.4. Response, Super-Response and Clinical Remission on Treatment

According to the EXACTO score, 95 patients (62.5%) achieved a complete response/super-response, 48 (31.6%) a good response and nine (5.9%) a partial response (Table 4). These classifications were based on the last available evaluable follow-up assessment for each patient. No patient was classified as a non-responder. The mean FEOS score was 78 ± 13 points, well above the proposed 70-point threshold for super-response [19]. Clinical remission on treatment, as defined by the modified international consensus criteria, was achieved by 95 patients (62.5%). Among patients with concomitant T2 comorbidities, complete remission of allergic rhinitis was observed in 82.2%, improvement of anosmia/hyposmia in 60% of those affected, and complete remission of atopic dermatitis in 51 out of 54 affected patients (94.4% of the affected subgroup).

### 3.5. Univariate and Multivariable Predictors of Complete Response (EXACTO = 1)

Patients reaching complete response had significantly higher baseline FEV1 (91.5 ± 15.7% vs. 67.1 ± 22.6%, *p* < 0.001), higher ACT (16.1 ± 2.2 vs. 15.1 ± 1.9, *p* = 0.003), higher blood eosinophil count (708 ± 606 vs. 450 ± 490 cells/µL, *p* = 0.005), and fewer OCS bursts in the previous year (3.2 ± 2.2 vs. 5.6 ± 5.1, *p* = 0.002), and were more likely to have allergic rhinitis (88.4% vs. 70.2%, *p* = 0.010) and CRSwNP (65.3% vs. 49.1%, *p* = 0.073) (Table 5).

The multivariable logistic regression model showed an apparent AUC of 0.91 and adequate calibration by the Hosmer–Lemeshow test. Independent positive predictors of complete response were a higher baseline FEV1% predicted (OR 1.10, 95% CI 1.06–1.14; *p* < 0.001), higher blood eosinophil count (OR 1.22 per 100 cell/µL, 95% CI 1.001–1.004; *p* = 0.002), the presence of allergic rhinitis (OR 8.85, 95% CI 1.93–40.55; *p* = 0.005), CRSwNP (OR 3.61, 95% CI 1.10–11.82; *p* = 0.034) and atopic dermatitis (OR 11.44, 95% CI 2.60–50.43; *p* = 0.001) (Table 6). Conversely, a higher number of prior-year OCS bursts and higher baseline FeNO were inversely associated with complete response in the multivariable model. However, the association with FeNO was not observed in the univariate analysis and should therefore be interpreted cautiously as an exploratory, adjusted association rather than as evidence of reduced dupilumab efficacy in patients with higher FeNO.

#### Subgroup Analysis According to Previous Biologic Exposure

Thirty patients (19.7%) had previously received another biologic agent, including benralizumab (n = 27), mepolizumab (n = 2) and omalizumab (n = 1). In an exploratory subgroup analysis, the overall response to dupilumab was comparable between patients with and without previous biologic exposure. The mean FEOS score was 77.8 ± 10.2 in previously biologic-exposed patients and 78.0 ± 13.7 in biologic-naïve patients (*p* = 0.898). Previously biologic-exposed patients showed greater improvements in ACT (+8.8 vs. +7.3 points; *p* = 0.001) and ACQ-5 (−4.03 vs. −3.56 points; *p* = 0.013), whereas no significant between-group differences were observed in FEV1 improvement (+300 vs. +316 mL; *p* = 0.305) or FeNO reduction (−45.1 vs. −30.3 ppb; *p* = 0.227). These findings should be considered exploratory given the small size of the previously exposed subgroup (Supplementary Material S2).

## 4. Discussion

**Figure 1:**
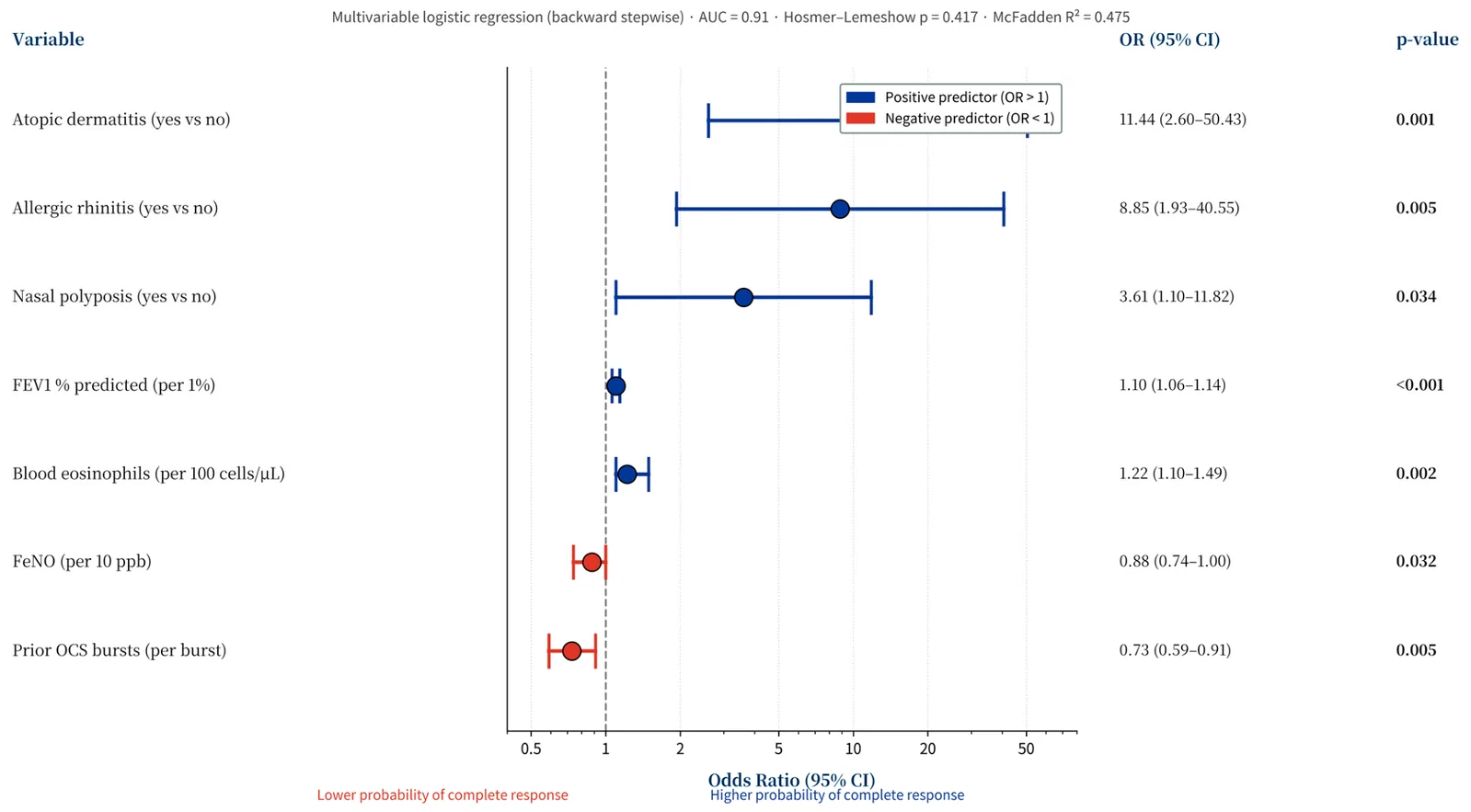
Forest plot of the multivariable logistic regression model for complete response (EXACTO = 1) after dupilumab treatment in 152 patients with severe uncontrolled asthma. Effect estimates are expressed as odds ratios (ORs) with 95% confidence intervals (CIs). Variables in blue (OR > 1) are independent positive predictors of complete response; variables in red (OR < 1) are negative predictors. The dashed vertical line marks OR = 1 (no effect). For continuous variables, ORs are reported per 1% increase (FEV1% predicted), per 100 cells/µL increase (blood eosinophils), per 10 ppb increase (FeNO) and per additional OCS burst, respectively. Model performance: AUC = 0.91; Hosmer–Lemeshow χ^2^ = 8.17, *p* = 0.417; McFadden’s pseudo R^2^ = 0.475; likelihood ratio *p* < 0.001.

**Figure 2:**
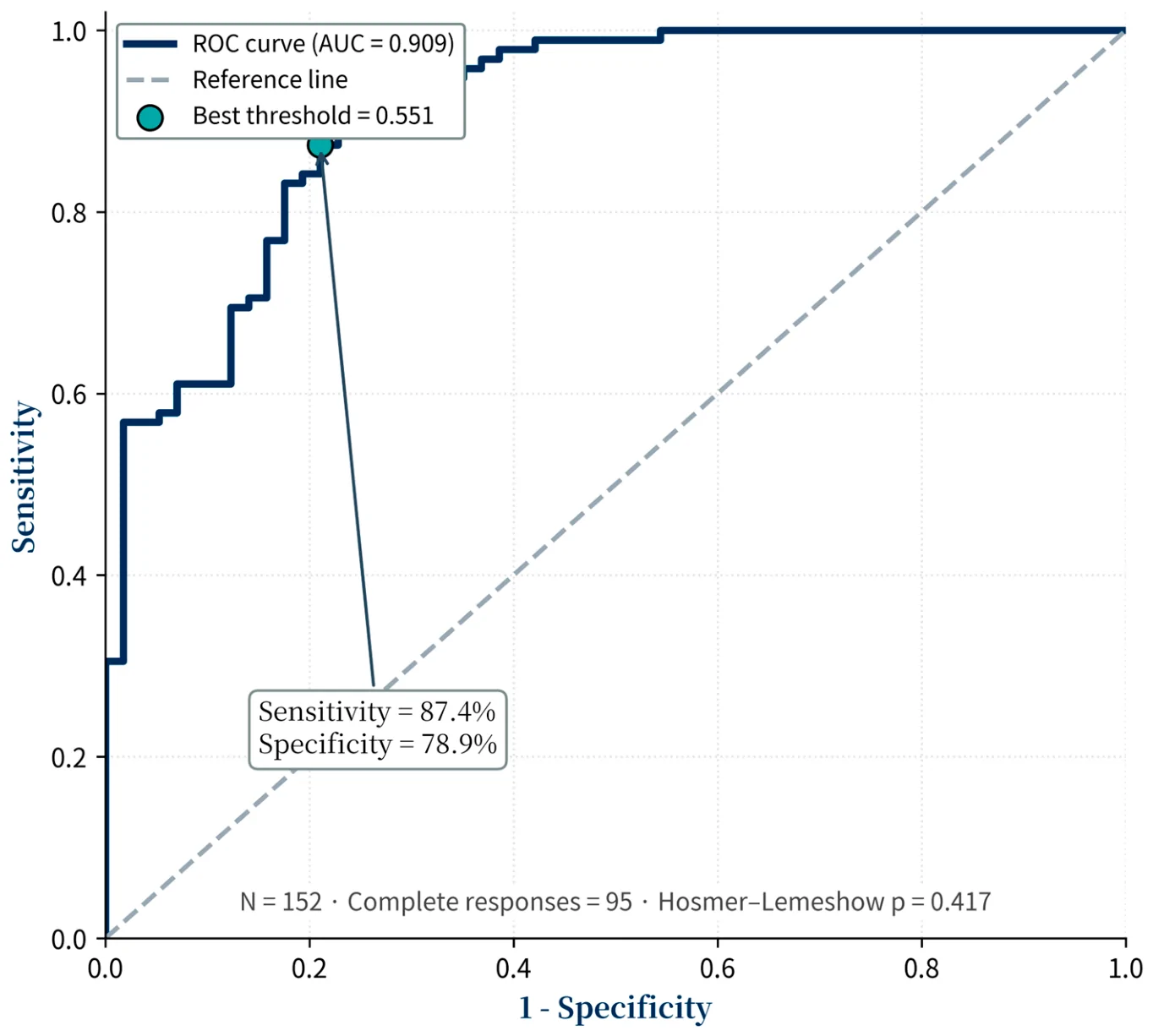
Receiver operating characteristic (ROC) curve of the final multivariable logistic regression model predicting complete response (EXACTO = 1) after dupilumab treatment. The model showed excellent discrimination, with an AUC of 0.909. The optimal cut-off probability according to the Youden index was 0.551, with sensitivity of 87.4% and specificity of 78.9%.

In this real-world cohort of 152 patients with severe uncontrolled asthma followed for nearly two years, dupilumab was associated with substantial improvements in asthma control, lung function, quality of life and healthcare resource utilisation. Clinical remission on treatment was observed in 62.5% of patients according to our predefined criteria. This proportion is within the range reported across real-world studies, although differences in remission definitions, follow-up duration and patient characteristics limit direct comparisons between cohorts. The main contribution of the present study is the longitudinal assessment of response using both the FEOS and EXACTO instruments, together with the evaluation of T2 comorbidities and baseline factors associated with complete response. Importantly, the multivariable model identified a profile of preserved baseline lung function and rich T2 signature (higher eosinophil count, allergic rhinitis, CRSwNP and atopic dermatitis) as the main driver of complete response, while previous heavy OCS exposure and very high baseline FeNO identified patients less likely to reach full remission, although still benefiting substantially (Figure 1 and Figure 2).

The magnitude of FEV1 improvement (+275 mL) and exacerbation reduction (−91%) observed in our cohort is in line with, and even exceeds, the results of the pivotal trials QUEST [10] and VENTURE [11]. Real-world cohorts have reported similar or slightly lower benefits: the French multicentre RAINBOW cohort (n = 297) [23] showed exacerbation reduction of 82%, ACT improvement of +6 and OCS withdrawal in 50–60% of dependent patients. The Italian SANI registry has also highlighted the clinical and demographic heterogeneity of patients with severe asthma, including relevant sex-related differences in disease characteristics [24] and the recent international systematic review and meta-analysis by Shackleford et al. [25] reported pooled remission rates of 30–50% at 12 months across biologics, with the highest values for dupilumab when applied to T2-rich phenotypes. The remission rate observed in our cohort should be interpreted in the context of the definition used and the variable duration of follow-up. Differences in patient phenotype, treatment duration and remission criteria may account for the variability in remission estimates reported across real-world cohorts. Therefore, direct comparisons with previously published cohorts should be made cautiously [3,20]. However, because follow-up duration was not identical for all patients, the possibility that longer observation increased the probability of fulfilling response or remission criteria cannot be excluded. Therefore, these remission estimates should be interpreted in the context of the variable follow-up duration and should not be directly equated with remission rates obtained at a fixed time point.

The strong association between T2 comorbidities and complete response observed in our multivariable model (Figure 1) is biologically plausible, since dupilumab acts on the shared IL-4/IL-13 axis driving all of them [10,11]. A recent multicentre Spanish cohort [26] identified the simultaneous presence of three or more T2 comorbidities as the strongest predictor of remission. The robust OCS-sparing effect observed in our patients (70.6% reduced and 58.8% completely withdrew OCS) is concordant with VENTURE (70%) [11] and with the recent SHAMAL study using benralizumab [27], reinforcing the call to introduce biologics early—before steroid-related comorbidity develops [17,18].

The dual benefit of dupilumab on upper-airway and cutaneous T2 comorbidities deserves particular emphasis: complete remission of allergic rhinitis was achieved in 82.2% of affected patients, improvement of anosmia/hyposmia in 60% of those affected, and complete remission of atopic dermatitis in 94.4% of the affected subgroup. These findings reflect the cross-organ activity of IL-4Rα blockade demonstrated in the LIBERTY AD CHRONOS [12] and SINUS-24/52 [13] pivotal programmes, and support the positioning of dupilumab as an effective biologic for patients with severe asthma and multiple coexistent T2 conditions.

The inverse association between higher baseline FeNO and the probability of complete response requires cautious interpretation. Although higher FeNO is generally associated with greater clinical benefit from dupilumab, particularly in terms of exacerbation reduction and lung function improvement, FeNO was not associated with complete response in our univariate analysis and showed only a modest inverse association after multivariable adjustment. This finding may reflect residual confounding, differences in the probability of achieving complete remission versus partial clinical benefit, or other characteristics of this real-world cohort. Therefore, it should be considered hypothesis-generating rather than interpreted as evidence that higher baseline FeNO predicts poorer response to dupilumab. Further studies are required to determine whether this association is reproducible.

Our study has several strengths: a sizeable real-world cohort, a relatively long follow-up with systematic clinical assessments, the simultaneous use of both FEOS and EXACTO scores, and a robust multivariable analysis with adequate internal validity (AUC = 0.91). Because the model was not internally resampled or externally validated, these performance estimates should be considered apparent and potentially optimistic. Limitations include the single-centre design, the absence of a control arm, the modest number of patients with maintenance OCSs (n = 18), which constrains the subgroup analysis of steroid sparing; the use of backward stepwise selection, which may increase the risk of overfitting and model instability, and the variability in individual follow-up duration inherent to the ambispective real-world design. Unequal follow-up may influence the probability of fulfilling response or clinical remission on treatment criteria, particularly when these outcomes are assessed at the last available visit rather than at a fixed time point. Although healthcare-resource outcomes were annualised, residual bias in outcome classification cannot be completely excluded. In addition, the apparently high and relatively uniform response rates observed in some outcomes warrant cautious interpretation and independent confirmation in larger multicentre cohorts with standardised assessment time points. An additional limitation concerns the potential mathematical coupling between some baseline predictors (FEV1, ACT, prior exacerbations and OCS use) and the domains incorporated into the EXACTO response definition. Therefore, the high discriminative performance of the predictive model should be interpreted cautiously, as part of the observed association may reflect overlap between predictor and outcome components rather than independent prognostic information. Finally, there was a lack of long-term assessment of remission stability after potential treatment de-escalation. Future multicentre studies with longer follow-up are needed to confirm whether complete responders sustain remission over time and whether biomarker-guided strategies based on the predictors identified here can be used to individualise patient selection and possible treatment de-escalation, as recently suggested in the literature [20,28].

## 5. Conclusions

In a real-life setting, dupilumab produced a profound and sustained improvement in asthma control, lung function, quality of life and healthcare resource utilisation in patients with severe uncontrolled asthma, allowing clinical remission on treatment in nearly two-thirds of patients and marked improvement of T2 comorbidities, with a favourable safety profile. Independent predictors of complete response were higher baseline FEV1, higher eosinophil count, allergic rhinitis, CRSwNP and atopic dermatitis, while higher prior OCS exposure and baseline FeNO showed inverse associations that should be interpreted cautiously.

## Figures and Tables

**Table 1:** Baseline demographic, clinical and laboratory characteristics.

Variable	Value (N = 152)
Age, years (mean ± SD)	50 ± 12
Female sex, n (%)	102 (67.1)
BMI, kg/m^2^ (mean ± SD)	26.4 ± 5.0
Smoking status, n (%)	
Current smokers, n (%)	6 (4)
Former smokers, n (%)	30 (20)
Never smokers, n (%)	116 (76)
Cumulative pack-years (mean ± SD)	6 ± 3
Asthma phenotype, n (%)	
Allergic early onset	24 (16.0)
Allergic late onset	28 (18.0)
Intrinsic with high IgE	12 (8.0)
Eosinophilic with CRSwNP	76 (50.0)
Eosinophilic without CRSwNP	12 (8.0)
Follow-up, months (mean ± SD)	21 ± 8
FEV1, L (mean ± SD)	2.44 ± 0.85
FEV1, % predicted (mean ± SD)	82.3 ± 22.0
Blood eosinophils, cells/µL (mean ± SD)	612 ± 577
Total IgE, IU/mL (mean ± SD)	1040 ± 3561
FeNO, ppb (mean ± SD)	55 ± 46
SNOT-22 (mean ± SD)	52.4 ± 33.9
ACT (mean ± SD)	15.7 ± 2.1
ACQ-5 (mean ± SD)	4.0 ± 0.9
VAS symptoms (mean ± SD)	8.2 ± 1.2
Mini-AQLQ (mean ± SD)	2.18 ± 0.36
Comorbidities (any), n (%)	124 (81.6)
Allergic rhinitis	124 (81.6)
Anosmia/hyposmia	102 (67.1)
CRSwNP	77 (50.7)
NSAID-exacerbated respiratory disease	56 (36.8)
Atopic dermatitis	54 (35.5)
Food allergy	20 (13.2)
Obesity	15 (9.9)
Allergic bronchopulmonary aspergillosis	8 (5.3)
Obstructive sleep apnoea	5 (3.3)
Bronchiectasis	3 (2.0)
Prior biologic, n (%)	30 (19.7)
Benralizumab	27 (17.8)
Mepolizumab	2 (1.3)
Omalizumab	1 (0.7)
Maintenance OCS, n (%)	18 (11.8)
OCS dose, mg/day prednisone-equivalent	7.0 ± 2.5
Hospital admission previous year, n (%)	55 (36.2)
ED visits previous year, n (%)	130 (85.5)
OCS bursts previous year, n (%)	137 (90.1)
Exacerbations/year (mean ± SD)	7.9 ± 5.5

Abbreviations: BMI, body mass index; FEV1, forced expiratory volume in 1 s; FeNO, fractional exhaled nitric oxide; SNOT-22, Sino-Nasal Outcome Test-22; ACT, Asthma Control Test; ACQ-5, Asthma Control Questionnaire-5; VAS, visual analogue scale; AQLQ, Asthma Quality-of-Life Questionnaire; CRSwNP, chronic rhinosinusitis with nasal polyps; NSAID, non-steroidal anti-inflammatory drugs; OCS, oral corticosteroid; ED, emergency department.

**Table 2:** Pre- and post-treatment comparison of efficacy outcomes (paired *t*-test).

Variable	Baseline (Mean ± SD)	Post-Treatment (Mean ± SD)	Δ	*p*-Value
FEV1, % predicted	82.4 ± 22.0	87.3 ± 20.0	+4.9	<0.001
FEV1, L	2.44 ± 0.85	2.75 ± 0.80	+0.275	<0.001
ACT score	15.7 ± 2.1	23.3 ± 1.4	+7.6	<0.001
ACQ-5 score	4.01 ± 0.91	0.35 ± 0.37	−3.65	<0.001
VAS symptoms (0–10)	8.21 ± 1.21	2.24 ± 0.91	−5.97	<0.001
Mini-AQLQ	2.18 ± 0.36	4.53 ± 0.37	+2.35	<0.001
SNOT-22	52.4 ± 33.9	15.9 ± 8.6	−36.6	<0.001
FeNO, ppb	55.3 ± 46.5	22.0 ± 15.1	−33.2	<0.001
Total IgE, IU/mL	1040.8 ± 3560.7	96.8 ± 203.2	−944	0.001
Blood eosinophils, cells/µL	611.5 ± 577.1	550.7 ± 470.8	−60.8	0.245
Exacerbations/year	7.9 ± 5.5	0.71 ± 1.98	−7.22	<0.001
ED visits/year	3.17 ± 2.85	0.28 ± 0.67	−2.89	<0.001
Hospital admissions/year	0.71 ± 1.38	0.08 ± 0.32	−0.63	<0.001
OCS bursts/year	4.06 ± 3.76	0.35 ± 0.93	−3.71	<0.001
Hospitalisation length (n = 11), days	17.5 ± 18.1	6.4 ± 3.2	−11.1	0.078
Maintenance OCS dose (n = 15), mg/day	7.00 ± 2.54	2.33 ± 2.58	−4.67	<0.001

Δ, mean difference (post−pre); FEV1, forced expiratory volume in 1 s; ACT, Asthma Control Test; ACQ-5, Asthma Control Questionnaire-5; VAS, visual analogue scale; AQLQ, Asthma Quality-of-Life Questionnaire; SNOT-22, Sino-Nasal Outcome Test-22; FeNO, fractional exhaled nitric oxide; ED, emergency department; OCS, oral corticosteroid.

**Table 3:** Concomitant medication and healthcare utilisation before vs. after dupilumab.

Variable	Baseline n (%)	Post n (%)	*p*-Value
Inhaled corticosteroids	152 (100)	152 (100)	1.000
Reduced ICS dose	—	50 (32.9)	—
LABA	151 (99.3)	149 (98.0)	0.500
SABA (regular use)	146 (96.1)	33 (21.7)	<0.001
LAMA/anticholinergic	78 (51.3)	57 (37.5)	0.002
Anti-leukotriene	99 (65.1)	69 (45.4)	<0.001
Anti-histamine	77 (50.7)	44 (28.9)	<0.001
Xanthines	7 (4.6)	2 (1.3)	0.063
Maintenance OCS	18 (11.8)	6 (3.9)	<0.001
OCS dose tapered (among 17 evaluable)	—	12 (70.6)	—
OCS withdrawn (among 17 evaluable)	—	10 (58.8)	—
≥1 hospital admission	55 (36.2)	11 (7.2)	<0.001
≥1 ED visit	130 (85.5)	20 (13.2)	<0.001
≥1 OCS burst	137 (90.1)	25 (16.4)	<0.001

McNemar test for paired binary variables. ICS, inhaled corticosteroid; LABA, long-acting β2-agonist; SABA, short-acting β2-agonist; LAMA, long-acting muscarinic antagonist; OCS, oral corticosteroid; ED, emergency department.

**Table 4:** Response, super-response, clinical remission on treatment and impact on T2 comorbidities.

Outcome	n (%)
EXACTO—complete response/super-response	95 (62.5)
EXACTO—good response	48 (31.6)
EXACTO—partial response	9 (5.9)
EXACTO—non-response	0 (0.0)
FEOS score, mean ± SD	78 ± 13
Clinical remission on treatment	95 (62.5)
Improvement of anosmia/hyposmia (among 102 affected)	61 (60.0)
Complete remission of allergic rhinitis (among 124 affected)	102 (82.2)
Partial improvement of allergic rhinitis	22 (17.7)
Complete remission of atopic dermatitis (among 54 affected)	51 (94.4)
Discontinuation of treatment	8 (5.3)
Adverse events—eosinophilic pneumonia	3 (2.0)
Adverse events—flu-like syndrome	1 (0.7)

**Table 5:** Univariate analysis of baseline factors associated with complete response (EXACTO = 1).

Variable	Complete Response (n = 95)	Other Response (n = 57)	OR (95% CI)	*p*-Value
Age, years	49.1 ± 11.0	52.4 ± 13.0	0.98 (0.95–1.01)	0.121
FEV1, % predicted	91.5 ± 15.7	67.1 ± 22.6	1.07 (1.05–1.10)	<0.001
ACT score	16.1 ± 2.2	15.1 ± 1.9	1.27 (1.08–1.51)	0.003
Prior-year OCS bursts	3.2 ± 2.2	5.6 ± 5.1	0.83 (0.74–0.93)	0.002
Blood eosinophils, cells/µL	708 ± 606	450 ± 490	1.001 (1.000–1.002)	0.005
FeNO, ppb	55.2 ± 45.8	55.4 ± 47.9	1.00 (0.99–1.01)	0.976
Allergic rhinitis, %	88.4	70.2	3.25 (1.39–7.57)	0.010
CRSwNP, %	65.3	49.1	1.95 (1.00–3.80)	0.073
Atopic dermatitis, %	40.0	28.1	1.71 (0.84–3.47)	0.189
Maintenance OCS at baseline, %	7.4	19.3	0.33 (0.12–0.92)	0.052

Continuous variables expressed as mean ± SD. OR per unit increase for continuous variables and OR for the presence of the condition for categorical variables. FEV1, forced expiratory volume in 1 s; ACT, Asthma Control Test; OCS, oral corticosteroid; FeNO, fractional exhaled nitric oxide; CRSwNP: chronic rhinosinusitis with nasal polyps.

**Table 6:** Multivariable logistic regression model for complete response (backward stepwise).

Variable	B	SE	OR (95% CI)	*p*-Value
(Intercept)	−9.708	1.998	—	<0.001
FEV1, % predicted (per 1%)	0.096	0.018	1.10 (1.06–1.14)	<0.001
Prior OCS bursts (per burst)	−0.313	0.110	0.73 (0.59–0.91)	0.005
Blood eosinophils (per 100 cell/µL)	0.002	0.001	1.22 (1.11–1.49)	0.002
FeNO (per 1 ppb)	−0.013	0.006	0.99 (0.97–1.00)	0.032
Allergic rhinitis	2.180	0.777	8.85 (1.93–40.55)	0.005
CRSwNP	1.283	0.605	3.61 (1.10–11.82)	0.034
Atopic dermatitis	2.437	0.757	11.44 (2.60–50.43)	0.001

Backward stepwise logistic regression (removal *p* > 0.10). Model fit: AUC = 0.91; McFadden pseudo R^2^ = 0.475; Hosmer–Lemeshow *χ*^2^ = 8.17, *p* = 0.417; LR *p* < 0.001. No multicollinearity (all VIF < 2). Reference category for binary variables = “no”. FEV1, forced expiratory volume in 1 s; OCS, oral corticosteroid; FeNO, fractional exhaled nitric oxide CRSwNP: chronic rhinosinusitis with nasal polyps.

## Data Availability

The data supporting the findings of this study are not publicly available because they contain potentially identifiable clinical information, but de-identified data may be made available by the corresponding author upon reasonable request and subject to institutional and ethical approval.

## Supplementary Materials

The following supporting information can be downloaded at https://www.mdpi.com/article/10.3390/jcm15166429/s1: Supplementary Material S1. STROBE checklist for the reporting of the present ambispective observational cohort study. The checklist indicates the corresponding sections of the manuscript in which each STROBE recommendation is addressed. Supplementary Material S2: Subgroup analysis according to previous biologic exposure.

## Abbreviations

The following abbreviations are used in this manuscript:

ACT	Asthma Control Test
ACQ-5	5-item Asthma Control Questionnaire
ATS	American Thoracic Society
AUC	Area Under the Receiver Operating Characteristic Curve
BMI	Body Mass Index
CEIm	Drug Research Ethics Committee
COPD	Chronic Obstructive Pulmonary Disease
CRSwNP	Chronic Rhinosinusitis with Nasal Polyps
ED	Emergency Department
ERS	European Respiratory Society
EXACTO	Multidimensional Response Score for Biologic Therapy in Severe Asthma
FEOS	FEV1, Exacerbations, Oral Corticosteroids and Symptoms Score
FeNO	Fractional Exhaled Nitric Oxide
FEV1	Forced Expiratory Volume in 1 Second
GEMA	Spanish Guideline for Asthma Management
GINA	Global Initiative for Asthma
ICS	Inhaled Corticosteroid
IgE	Immunoglobulin E
IL-4	Interleukin-4
IL-4Rα	Interleukin-4 Receptor Alpha
IL-13	Interleukin-13
LABA	Long-Acting β2-Agonist
LAMA	Long-Acting Muscarinic Antagonist
MCID	Minimal Clinically Important Difference
NERD	NSAID-Exacerbated Respiratory Disease
NSAID	Non-Steroidal Anti-Inflammatory Drug
OCS	Oral Corticosteroid
OR	Odds Ratio
PRO	Patient-Reported Outcome
ROC	Receiver Operating Characteristic
SABA	Short-Acting β2-Agonist
SD	Standard Deviation
SNOT-22	22-item Sino-Nasal Outcome Test
SUA	Severe Uncontrolled Asthma
T2	Type 2 (Inflammation)
VAS	Visual Analogue Scale
