# Supplementary material for: Real-World Effectiveness of Dupilumab in Severe Uncontrolled Asthma: Clinical Remission on Treatment, Predictors of Complete Response and Impact on Type-2 Comorbidities
Source: J Clin Med. 2026 Aug 20;15(16):6429. doi: 10.3390/jcm15166429 (PMC13513868; doi:10.3390/jcm15166429)
Supplement: Supplementary file 1 [file jcm-15-06429-s001.zip › jcm-4457073-supplementary.pdf]

***Supplementary Material S1**. STROBE checklist for the reporting of the present ambispective observational cohort study. The checklist indicates the corresponding sections of the manuscript in which each STROBE recommendation is addressed.*

**Item**	**Recommendation**	**Reported in manuscript**	**Location**
Title and abstract	Indicate the study's design with a commonly used term in the title or abstract.	The abstract describes the study as a real-world cohort and reports the observational setting.	Abstract
1	Provide an informative and balanced summary of background, methods, results, and conclusions.	Background, Methods, Results and Conclusions are presented in the abstract, including study design limitations.	Abstract
2	Explain the scientific background and rationale for the investigation.	The rationale for evaluating dupilumab's effectiveness, remission and predictors of complete response is described.	Introduction
3	State specific objectives, including any pre-specified hypotheses.	The study objective is to evaluate real-world effectiveness and factors associated with complete response.	Introduction
4	Present key elements of study design early in the paper.	The ambispective observational cohort design is described in the Methods.	Methods
5	Describe setting, locations, and relevant dates, including recruitment, exposure, follow-up, and data collection.	The single-centre setting, study period and follow-up are described.	Methods
6	Give eligibility criteria and sources/methods of selection of participants.	Eligibility criteria and patient selection are described.	Methods
7	Clearly define outcomes, exposures, predictors, potential confounders, and effect modifiers.	Clinical response/remission, treatment exposure, baseline characteristics and predictors are defined.	Methods
8	For each variable of interest, give sources of data and details of methods of assessment.	Clinical, functional, biomarker and patient-reported outcomes are described, including relevant instruments.	Methods
9	Describe any efforts to address potential sources of bias.	The manuscript acknowledges the uncontrolled design, variable	Methods; Discussion

		follow-up, potential overfitting and mathematical coupling.	
10	Explain how the study size was arrived at.	The cohort consists of all eligible patients treated and followed at the study centre during the predefined study period.	Methods
11	Explain how quantitative variables were handled in the analyses and which groupings were chosen.	Continuous variables and categorical variables are described, with statistical methods specified.	Statistical analysis
12	Describe all statistical methods, including methods used to control for confounding, assess subgroups/interactions, handle missing data, and address sensitivity analyses.	Descriptive and comparative analyses, univariable screening and multivariable logistic regression are described. Exploratory subgroup analysis is reported. Limitations of model validation are acknowledged.	Statistical analysis; Results; Discussion
13	Report numbers of individuals at each stage of study, reasons for non-participation, and a flow diagram if appropriate.	The number of included patients and relevant subgroup sizes are reported; patient selection is described.	Methods; Results
14	Give characteristics of study participants and information on exposures and potential confounders.	Baseline demographic, clinical, treatment and biomarker characteristics are presented in Table 1.	Results; Table 1
15	Report numbers of outcome events or summary measures over time.	Treatment outcomes, changes from baseline and healthcare-resource outcomes are reported.	Results; Table 2
16	Give unadjusted estimates and, if applicable, confounder-adjusted estimates and precision.	Unadjusted comparisons and multivariable odds ratios with 95% confidence intervals are reported for the predictor model.	Results; Table 3
17	Report category boundaries when continuous variables were categorized.	Categorization is described where applicable; continuous variables are otherwise retained as continuous measures.	Methods
18	Translate relative risks into absolute risks for a meaningful time period when appropriate.	Treatment-response proportions and absolute changes are reported; relative risk conversion is not applicable to the principal analysis.	Results

19	Report other analyses done, e.g., subgroup analyses and sensitivity analyses.	An exploratory subgroup analysis according to previous biologic exposure is reported.	Results
20	Summarize key results with reference to study objectives.	The principal effectiveness and predictor findings are summarized.	Results; Discussion
21	Discuss limitations, including sources of potential bias or imprecision.	Single-centre design, absence of a control arm, variable follow-up, subgroup size, potential overfitting, mathematical coupling and lack of external validation are discussed.	Discussion
22	Give a cautious overall interpretation considering objectives, limitations, multiplicity of analyses, results from similar studies, and other relevant evidence.	Findings are interpreted cautiously in the context of real-world cohorts and existing literature.	Discussion
23	Discuss generalisability/external validity of the study results.	The need for independent confirmation in larger multicentre cohorts is explicitly acknowledged.	Discussion
24	Give the source of funding and the role of funders for the present study and, if applicable, for the original study.	The manuscript states that there was no external funding.	Funding

Supplementary Material S2: *Subgroup analysis according to previous biologic exposure.*

Outcome	Previous biologic (n=30)	Biologic-naïve (n=122)	p
**FEOS score**	77.8 ± 10.2	78.0 ± 13.7	0.898
**Δ ACT**	+8.8	+7.3	0.001
**Δ ACQ-5**	−4.03	−3.56	0.013
**Δ FEV1, mL**	+300	+316	0.305
**Δ FeNO, ppb**	−45.1	−30.3	0.227
**Δ SNOT-22**	−44.0	−34.7	0.094
**Δ mini-AQLQ**	+2.62	+2.29	0.002
